# miRNA expression profile changes in the peripheral blood of monozygotic discordant twins for epithelial ovarian carcinoma: potential new biomarkers for early diagnosis and prognosis of ovarian carcinoma

**DOI:** 10.1186/s13048-020-00706-8

**Published:** 2020-08-27

**Authors:** Seref Bugra Tuncer, Ozge Sukruoglu Erdogan, Seda Kilic Erciyas, Mukaddes Avsar Saral, Betul Celik, Demet Akdeniz Odemis, Gozde Kuru Turkcan, Hulya Yazici

**Affiliations:** Department of Cancer Genetics, Istanbul Faculty of Medicine, Oncology Institute, Istanbul University, Istanbul, Turkey

**Keywords:** Monozygotic twins, miRNA expression profiles, BRCA1 and BRCA2, Biomarkers

## Abstract

**Background:**

Ovarian cancer is the second most common gynecologic cancer with high mortality rate and generally diagnosed in advanced stages. The 5-year disease-free survival is below 40%. MicroRNAs, subset of the non-coding RNA molecules, regulate the translation in post transcriptional level by binding to specific mRNAs to promote or degrade the target oncogenes or tumor suppressor genes. Abnormal expression of miRNAs were found in numerous human cancer, including ovarian cancer. Investigating the miRNAs derived from the peripheral blood samples can be used as a marker in the diagnose, treatment and prognosis of ovarian cancer. We aimed to find biological markers for early diagnosis of ovarian cancer by investigating BRCA1 gene mutation carrier monozygotic discordant twins and their high risk healthy family individual’s miRNAs.

**Methods:**

The study was conducted on monozygotic twins discordant for ovarian cancer, and the liquid biopsy exploration of miRNAs was performed on mononuclear cells that were isolated from the peripheral blood samples. The miRNA expression profile changes in the study were found by using microarray analysis. miRNA isolation procedure performed from the lymphocyte in accordance with the kit protocol. The presence and quality of the isolated miRNAs screened by electrophoresis. Raw data logarithmic analysis was studied by identifying the threshold, normalization, correlation, mean and median values. Target proteins were detected for each miRNA by using different algorithms.

**Results:**

After the comparison of monozygotic discordant twins for epithelial ovarian carcinoma upregulation of the 4 miRNAs, miR-6131, miR-1305, miR-197-3p, miR-3651 and downregulation of 4 miRNAs, miR-3135b, miR-4430, miR-664b-5p, miR-766-3p were found statically significant.

**Conclusions:**

The detected 99 miRNAs out of 2549 miRNAs might be used in the clinic as new biological indicators in the diagnosis and follow up of epithelial ovarian cancer with complementary studies. The *miRNA expression* profiles were identified to be statistically significant in the evaluation of ovarian cancer etiology, BRCA1 mutation status, and ovarian cancer risk in accordance with the obtained data.

There is a need for validation of the miRNAs which were particularly detected between monozygotic twins and its association with ovarian cancer was emphasized in our study in wider cohorts including ovarian cancer patients, and healthy individuals.

## Background

Ovarian cancer is a significant cause of mortality in gynecologic cancers and one of the leading cause of cancer-associated mortality [1]. In Turkey, ovarian cancer is the 7th most common type of cancer in women in accordance with worldwide. Globocan 2018 data’s show that each year more than 295.000 women are diagnosed with ovarian cancer (OC) worldwide, and approximately 185.000 women die from it. The data of Globocan 2018 for Turkey shows that annually 3729 women are diagnosed with ovarian cancer, and 2191 women die from this malignancy. The 5-year survival rate was given as 23.8%. These data revealed that ovarian cancer is an important reason of gynecologic cancer associated mortality rate [2]. The epithelial ovarian cancers (EOC) originating from the ovarian surface epithelium constitutes approximately 90% of ovarian malignancy [3]. The majority (70%) of EOC patients are diagnosed in advanced stages (Stage III, and IV), and 5 year free-survival rate is below 40% [4]. The standard treatment for newly diagnosed ovarian cancer is the combination of cytoreductive surgery and platin-based chemotherapy. Significant advances in radical surgery and chemotherapy strategies have improved clinical outcomes, but unfortunately, no progress has been made with relapse and treatment resistance [5]. Ninety percent of ovarian cancer occurs sporadically in the population, whereas hereditary type appears 10% of ovarian cancer patients. BRCA1 and BRCA2 genes are the most common breast-ovarian cancer syndrome associated genes. Both BRCA1 and BRCA2 have roles in the control of the genomic stability, cell cycle, and apoptosis. The mutations occurring in these genes result with the inability of DNA repair, and therefore results in the accumulation of the mutations in the cell. The rate of the breast cancer susceptibility of women with BRCA1 gene mutation until the age of 80 years was 72% and the rate of ovarian cancer susceptibility rate is 44%, breast cancer susceptibility women with BRCA2 gene mutation until the age of 80 years is 69% and ovarian cancer development risk is 17% [6]. Twin studies became important on genetics by the end of the nineteenth century. Genetic and epidemiologic studies with monozygotic twins were accepted as highly useful investigation models in the past decades and have been used recently [7]. When a similarity for a disease or a quantitative feature between monozygotic and dizygotic twins is compared, variations are excluded according to studies conducted in the population and therefore, it is easier to identify and make etiological differences visible via twin studies. Because the affected siblings and dizygotic twins share the (approximately) 50% of the differentiated genes, the phenotypic differences between twins are known to be associated with the genetic variation. In addition, diversity may be revealed with a very limited patient population. Therefore, the results of the twin studies can be applied to the population and can make valuable contributions to the genetic studies. Monozygotic twins are genetically similar and generally expected to be compatible for congenital malformations, chromosomal abnormalities and Mendelian disorders. There are numerous studies conducted via discordant monozygotic twins revealing the genetic contribution [8]. Therefore, investigating the genetic variability in monozygotic twins is highly important and the majority of the human genetics associated research focus on finding genetic variability in discordant monozygotic twins. Phenotypically discordant monozygotic twins are used as the model systems in identification of the variable in understanding the pathogenesis of a disease. The most striking study is the one conducted with monozygotic twins in Canada and evidencing that multiple sclerosis (MS) was a genetic disease [9]. MicroRNAs are one of the subset of the non-coding RNAs generally consisting of single strand in 19–24 nucleotide length, not transformed to protein, having roles in post transcriptional regulation or suppression of translation of the target mRNAs [10, 11]. The regulatory roles of miRNAs were demonstrated to occur in tumorigenesis, cell differentiation, proliferation and apoptosis [12–15]. miRNA genes are known to locate in the chromosomal breaks. This DNA breaks cause chromosomal abnormalities frequently associated with cancer susceptibility and tumor development [16–18]. The non-invasive biological indicators have been used for the treatment resistance of ovarian cancer. The most common of this indicators are the cancer antigen-125 (CA-125) and cancer antigen-15-3 (CA-15-3). These biological indicators can be used in the follow-up of the treatment response in the diagnosed patients but cannot be used in the early diagnosis and in differentiation of the malignant disease [19]. Therefore, there is a need for special therapeutic agents customized for patients that may be used target specific therapies and in the early diagnosis of the ovarian cancer in identification of the efficacy of therapy and in the follow up period. Thus, studies investigating the target molecules and biological indicators are required that will enable the early diagnosis and in the development of the better therapy options. Differentially synthesize miRNAs such as miR − 200, miR-141, miR-125b, miR-222-3p or let-7 family has been shown in studies with ovarian cancer patients [20]. However, the use of these miRNAs as a biomarker in ovarian cancer is not yet available. In order to clearly define the role of miRNAs in the pathogenesis of ovarian cancer, we planned to investigate the BRCA mutant monozygotic twins with the same genetic profile but with discordant for ovarian malignant transformation. In this study, 2549 miRNAs, which are thought to have the potential biological indicator role, were studied from blood samples of both discordant monozygotic twins and BRCA wild type healthy siblings.

## Methods

### Patients recruitment

**Fig. 1:**
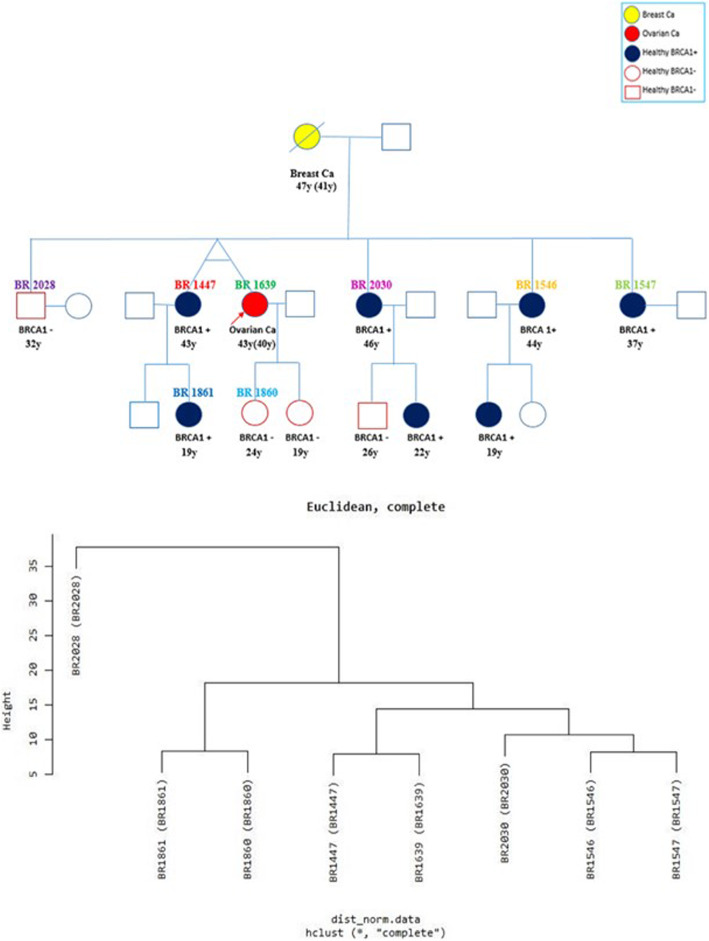
The pedigree of the family included in the study and their hierarchical cluster analysis. Legend: The pedigree of the family and using the correlations between samples, plotted a dendrogram for sample grouped by Hierarchical clustering. (Euclidean distance, Complete Linkage) BR 2028: Healthy Brother, BRCA1 negative (non-BRCA1 mutation carrier); BR 1861: Healthy Niece, BRCA1 positive (BRCA1 mutation carrier); BR 1860: Healthy Daughter, BRCA1 negative; BR 1447: Healthy Monozygotic twin, BRCA1 positive (BRCA1 mutation carrier); BR 1639: Monozygotic twin diagnosed with ovarian cancer, BRCA1 positive (BRCA1 mutation carrier); BR 2030: Healthy Sister, BRCA1 positive (BRCA1 mutation carrier); BR 1546: Healthy Sister, BRCA1 positive (BRCA1 mutation carrier); BR 1547: Healthy Sister, BRCA1 positive (BRCA1 mutation carrier)

The peripheral blood lymphocytes of monozygotic twins, discordant for ovarian cancer and healthy individuals in the same family were used in the study. The patient diagnosed with ovarian cancer and all family members, applied to the Cancer Genetics Clinic of Oncology Institute of Istanbul University for BRCA (breast cancer susceptibility) gene testing were examined for BRCA gene mutation. All family members in the study consisted of high-risk individuals with Hereditary Breast and Ovarian Cancer (HBOC) syndrome and the people included in the study were given as BR codes according to patient file number. The monozygotic ovarian cancer patient, healthy monozygotic twin, healthy 3 sisters, and 1 niece were found to have BRCA1 gene mutation, c.5266 dupC p.Gln1756Profs*74 rs397507247 on exon 20. The patient’s brother and daughter were found negative for BRCA1/BRCA2 gene mutations. In this study, lymphocyte cells separated from peripheral blood belonging to total of 8 cases including younger age ovarian cancer patient and healthy monozygotic twin, a patient’s daughter, 2 elder sisters, a younger sister, a nephew and a brother were examined by miRNA microarray method. The pedigree of the family included in the study and their hierarchical cluster analaysis via Euclidean method is shown on Fig. 1.

The study was approved by the Ethics Committee of the Istanbul Faculty of Medicine. Following Institutional Ethics Committee approval, informed consents were obtained from all participants before enrollment into the study (Ethics Committee Approval Number: 2016/4).

### Lymphocyte and miRNA isolation

Ficoll (Sigma-Aldrich, Darmstadt, Germany) density gradient was used to separate white blood cells (mononuclear cells) from other blood components. miRNA isolation procedure was performed from the lymphocyte in accordance with the kit protocol using the miRNeasy Mini Kit (Qiagen, cat No./ID: 217004). The procedure steps in accordance with the protocol are as follows; 700 μL QIAzol solution was included on the cells stored in nitrogen tank. Cell fractionation was enabled by mixturing using the vortex. For complete nucleoprotein fractionation they were stored at 24 °C room temperature for 5 min. By adding 140 μL chloroform, they were shacked and mixed on hand. The tubes were incubated for 2–3 min at 24 °C room temperature, then were centrifuged for 15 min at + 4 °C, and 12.000 g. The supernatant formed after centrifugation was transferred to collection tube using a pipette and was mixed using vortex by inclusion of 525 μL 100% ethanol. The supernatant formed after the ethanol centrifuging was transferred to collection tube was removed with a pipette, was mixed with vortex by including 525 μL %100 ethanol. Seven hundred microliters was taken from the obtained mixture and was transferred to the RNeasy MiniElute spin colon placed on 2 mL collection tube. The tubes were centrifuged for 15 s at 8000 g at 24 °C room temperature. Seven hundred microliters RWT buffer was added to spin colons and the colons were washed by centrifuging at 8000 g for 15 s.

The centrifuging procedure was repeated by including 500 μL RPE buffer twice consecutively to colons. The colons placed into clean tubes with 2 mL were dried by centrifuging 1 min in maximum speed. The colons placed in 1.5 mL sterile tubes were included 50 μL distilled water by centrifuging at 8000 g in 1 min and the miRNAs were collect.

### The quality control of the miRNAs

The presence and quality of the isolated miRNAs were screened by electrophoresis at 150 V on 1.5 Agarose gel. Then, the purity and concentrations of the miRNAs were measured on Thermo Scientific NanoDrop 2000 (spectrophotometer NanoDrop Technologies, Wilmington, DE, USA) device. The miRNA purity for each person in accordance with the NanoDrop device measurement result were obtained with the comparison of the measurements at spectrophotometrically at 260 nm and 280 nm wave lengths. The measurement rates at 260/280 nm wave lengths is a sign of quality of the purity of the samples, therefore the samples in the ideal value interval of 1.8 and 2.2 for RNA measurements were included in the study. The purity of miRNAs were evaluated using a Bioanalyser device (2100 Bioanalyser, Agilent Technologies, Santa Clara, CA, USA), Agilent RNA 6000 Nano Kit (Agilent Technologies, Santa Clara, CA, USA) for confirming whether the miRNAs were appropriate and in adequate level for microarray analysis. The evaluated sample concentrations and results were analyzed. The samples with RNA concentrations between 100 ng/μL and rRNA rate over 1 and RNA integrity number values between 7 and 9 were evaluated as the appropriate samples for array study.

### Microarray trial protocol

Microarray protocol was performed by preparing the Spike-in solution, sample marking, hybridization, sample dephosphorylation, sample denaturation, sample ligation, hybridization of the samples, slide loading, preparation of the hybridization unit and elution and scanning of slides. The slide scanning procedure was performed using the Agilent Microarray Scanner (Agilent Microarray Scanner with Surescan High Resolution Technology, Agilent Technologies, Santa Clara, CA, USA) device. The scanning procedure of the slides were performed on SurePrint G3 Human miRNA Microarray, Release 21.0, 8x60K (Agilent, Inc. Santa Clara, CA) platform, and using the Agilent Technologies G2600D scanning protocol. The analysis of the “TIFF’ (Tagged Image File Format) extensioned files obtained after scanning procedure was performed using the Agilent Feature Extraction v11.0.1.1 program.

The success levels of stages developed in all experiment process with this analysis program, the quality of the levels, the process were monitored and evaluated. Then, Bioinformatic Analysis procedure was performed.

### Data analysis

Raw data logarithmic analysis was studied by identifying the threshold, normalization, correlation, mean and median values. Then, the miRNAs demonstrating different expression profile among the samples were filtered. Using the Fold & change rates and independent two sample T test, the possible difference between the compared groups were evaluated. All evaluations were performed to enable the cut-off values as the fold&change rates |FC| ≥ 2, and *p*-value < 0.05. Hierarchical cluster analysis was performed using the Euclidean method (Fig. 1) and Complete Linkage cluster method. The control of the experimental errors and the detection of the erroneous finding rate were identified using the Hochberg method.

### Bioinformatic analysis

Target proteins were detected for each miRNA by using two algorithms, Targetscan7.1 (https://www.targetscan.org/vert_71/) and MirdbV5 (https://mirdb.org/miRDB/).

The targeted genes thought for each miRNA were confirmed by also both algorithms and the miRNA-target relations were also experimentally confirmed mirTarbase7.0 (https://mirtarbase.mbc.nctu.edu.tw/php/index.php) database.

### Comparison groups

In the study, miRNA analysis was performed at the genome level with/without mutation in cases with/without ovarian cancer. The miRNA data was evaluated by comparing different groups in order to investigate the effect of BRCA mutation in ovarian malignancy development and determine the miRNAs that can be important in the ovarian cancer pathogenesis: In Group 1; the monozygotic twins discordant for ovarian cancer were compared in order to find the effects of miRNAs in the formation of ovarian cancer. In Group 2; the family members with BRCA1 mutation were compared with family members without BRCA1 mutation to identify the changes of miRNAs expression levels according to BRCA positivity. In Group 3; the monozygotic ovarian cancer patient with BRCA1 mutation carrier and the other healthy family members with mutation carrier were compared for investigate the effects of both ovarian cancer development and BRCA positivity on miRNAs expression level. In Group 4; all family members were compared with ovarian cancer monozygotic twin in order to find the miRNAs that might be important in the predisposition of ovarian cancer. The comparison groups also showed in Table 1.

**Table 1:** Comparison groups and cases in the groups

	Group 1	Group 2	Group 3	Group 4
**Case**	BR1639	BR1639 BR1447 BR1547 BR2030 BR1546 BR1861	BR1639	BR1639 BR1447
**Control**	BR1447	BR1850 BR2028	BR1447 BR1547 BR2030 BR1546 BR1861	BR1547 BR2030 BR1546 BR1861 BR1850 BR2028

## Results

**Fig. 2:**
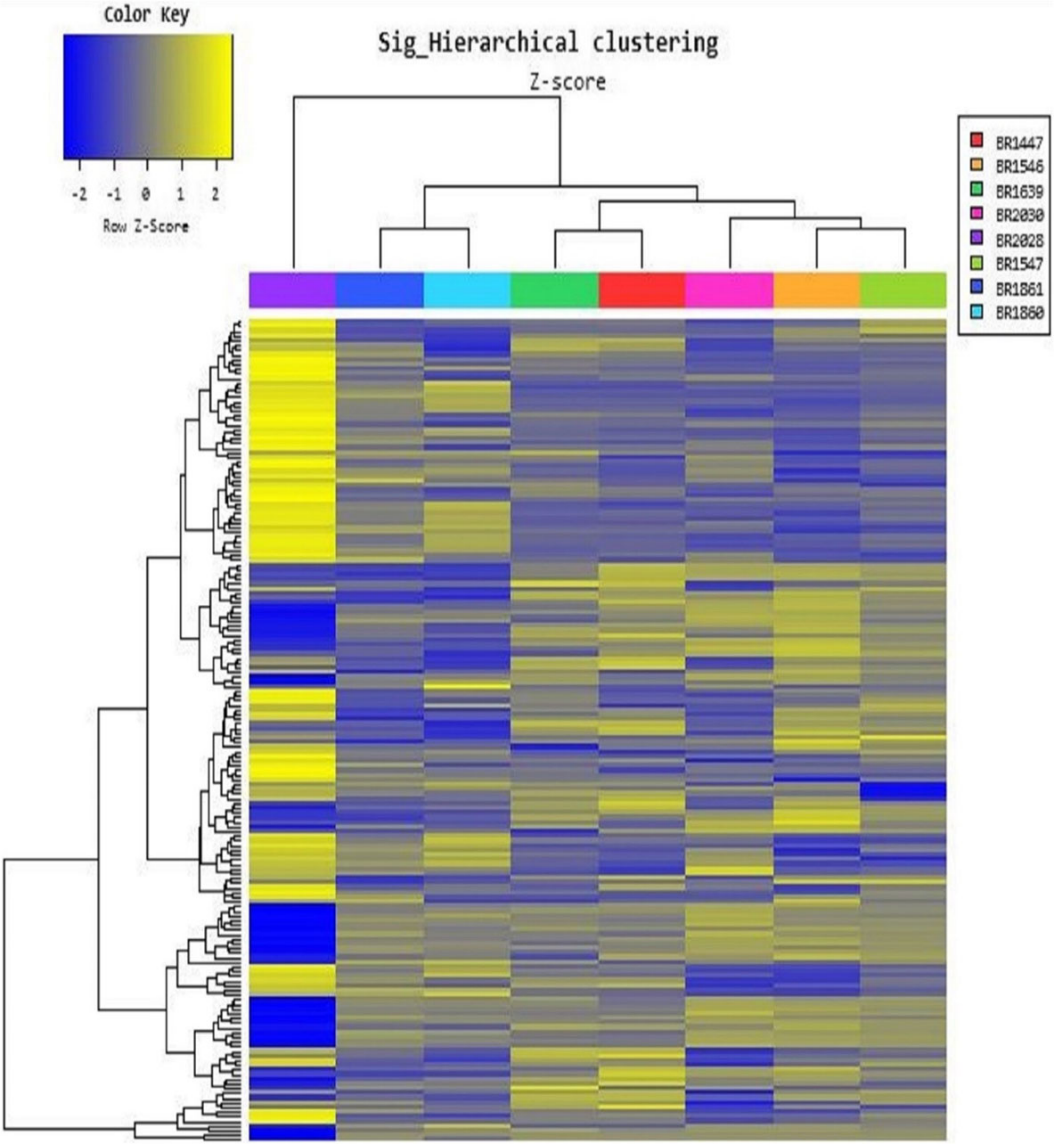
Hierarchical cluster analysis of the expression of 99 miRNAs. Legend: BR 2028: Healthy Brother, BRCA1 negative (non-BRCA1 mutation carrier); BR 1861: Healthy Niece, BRCA1 positive (BRCA1 mutation carrier); BR 1860: Healthy Daughter, BRCA1 negative; BR 1447: Healthy Monozygotic twin, BRCA1 positive; BR 1639: Monozygotic twin diagnosed with ovarian cancer, BRCA1 positive; BR 2030: Healthy Sister, BRCA1 positive; BR 1546: Healthy Sister, BRCA1 positive; BR 1547: Healthy Sister, BRCA1 positive. The miRNAs that may be effective in the etiology of ovarian cancer were identified after the comparison of monozygotic twins who were phenotypically discordant for ovarian cancer diagnosis in group 1

We identified 2549 differentially expressed comparison of miRNAs between the groups. The raw data obtained after experimental studies were filtered before the comparisons between the groups. The upregulated or downregulated miRNAs expression levels more than 2 fold (FC > 2) and smaller than the *p* value 0.05 (*p* < 0.05) were considered in evaluation and the comparisons between the groups were performed based on these values. All these comparisons were evaluated for ovarian cancer etiology, BRCA1 mutation carriage and the ovarian cancer risk. Hierarchical cluster analysis of the expression of 99 miRNAs represents sharp separations of up-regulated (yellow) from down-regulated (blue) in Fig. 2.

**Fig. 3:**
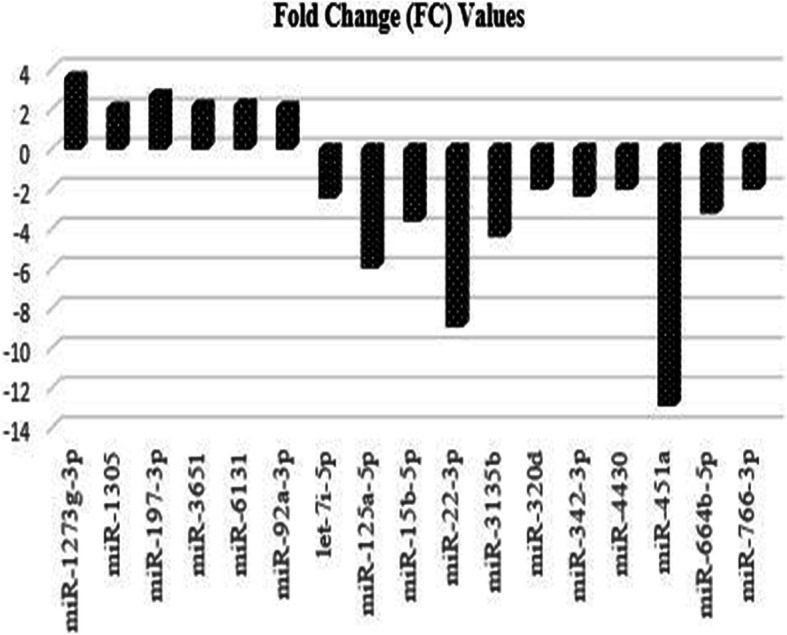
The upregulated and downregulated miRNAs and fold&changes associated with ovarian cancer etiology in monozygotic twins

17 miRNAs total of 2549 miRNAs were found statistically different after the comparison of phenotypically discordant monozygotic twin siblings. The 6 miRNAs miR-1273 g-3p, miR-1305, miR-197-3p, miR-3651, miR-6131, and miR-92a-3p expressions were found to have upregulated, and the other 11 miRNAs, let-7i − 5p, miR-125a-5p, miR-15b-5p, miR-22 − 3p, miR-3135b, miR-320d, miR-342-3p, miR-4430, miR-451a, miR-664b-5p, and miR-766-3p expressions were found to have downregulated. After the bioinformatic analysis, a total of 17 upregulated and downregulated statistically significant miRNAs and their target molecules are given in Table 2 and Fig. 3.

**Table 2:** Ovarian cancer etiology related upregulated and downregulated miRNAs and target proteins in monozygotic twins

miRNAs	Fold change (FC) values	Sequence of miRNA	miRNAStatus	Target genes
miR-1273 g-3p	3,57	ACCACUGCACUCCAGCCUGAG	Upregulated	*ZNF138, TMEM239, BMP3*
miR-1305	2,05	UUUUCAACUCUAAUGGGAGAGA	Upregulated	*PTPN4, PRKAA1, PAPD7,* *FRAT2, DEPDC1, FBXO41*
miR-197-3p	2,7	UUCACCACCUUCUCCACCCAGC	Upregulated	*CD82, PMAIP1, MTHFD1, CHECK1, AGO1, CASP10*
miR-3651	2,17	CAUAGCCCGGUCGCUGGUACAUGA	Upregulated	*RACGAP1, OLA1, TEX261,* *PTGS1, NFIC, ZNF200*
miR-6131	2,22	GGCUGGUCAGAUGGGAGUG	Upregulated	*PAGR1, IGF2BP1, CACNG8*
miR-92a-3p	2,09	UAUUGCACUUGUCCCGGCCUGU	Upregulated	*STAT3, PTEN, ATM, NOTCH2, CDH1, NFKB1*
let-7i-5p	−2,45	UGAGGUAGUAGUUUGUGCUGUU	Downregulated	*TLR4, BMP4, EIF2C1, NEUROG1, SOCS1, IGF1*
miR-125a-5p	-5,96	UCCCUGAGACCCUUUAACCUGUGA	Downregulated	*ERBB3, CDKN1A, TP53, ERBB2, EGFR, STAT3,MYC, VEGFA*
miR-15b-5p	-3,61	UAGCAGCACAUCAUGGUUUACA	Downregulated	*BCL2, VEGFA, CCND1, CCNE1, CDK1, CDK4, CDK6, E2F3, MAPK1*
miR-22-3p	-8,92	AAGCUGCCAGUUGAAGAACUGU	Downregulated	*CDKN1A, WNT1, ERBB3, MYCBP, HMGB1, E2F2, PTEN, POTED, SOD2,*
miR-3135b	-4,38	GGCUGGAGCGAGUGCAGUGGUG	Downregulated	*BIRC5, ABL2, MAPK1, MYCN*
miR-320d	-2,01	AAAAGCUGGGUUGAGAGGA	Downregulated	*DCTN5, SYNCRIP, FBXO28*
miR-342-3p	-2,36	UCUCACACAGAAAUCGCACCCGU	Downregulated	*GEMIN4, DNMT1, ID4, SREBF1,SREBF2, BMP7*
miR-4430	-2,01	AGGCUGGAGUGAGCGGAG	Downregulated	*ZNF485,ABL2, MAPK1, MSH5, PTEN*
miR-451a	-12,89	AAACCGUUACCAUUACUGAGUU	Downregulated	*CPNE3, RAB5A, IL6R, AKT1, MMP2*
miR-664b-5p	-3,21	UGGGCUAAGGGAGAUGAUUGGGUA	Downregulated	*CD55,MSN, RHOBTB3, PLAG1*
miR-766-3p	-2,02	ACUCCAGCCCCACAGCCUCAGC	Downregulated	*COX1, MAPK1, NF2, RAD51, STK4, STK24, VEGFC*

**Fig. 4:**
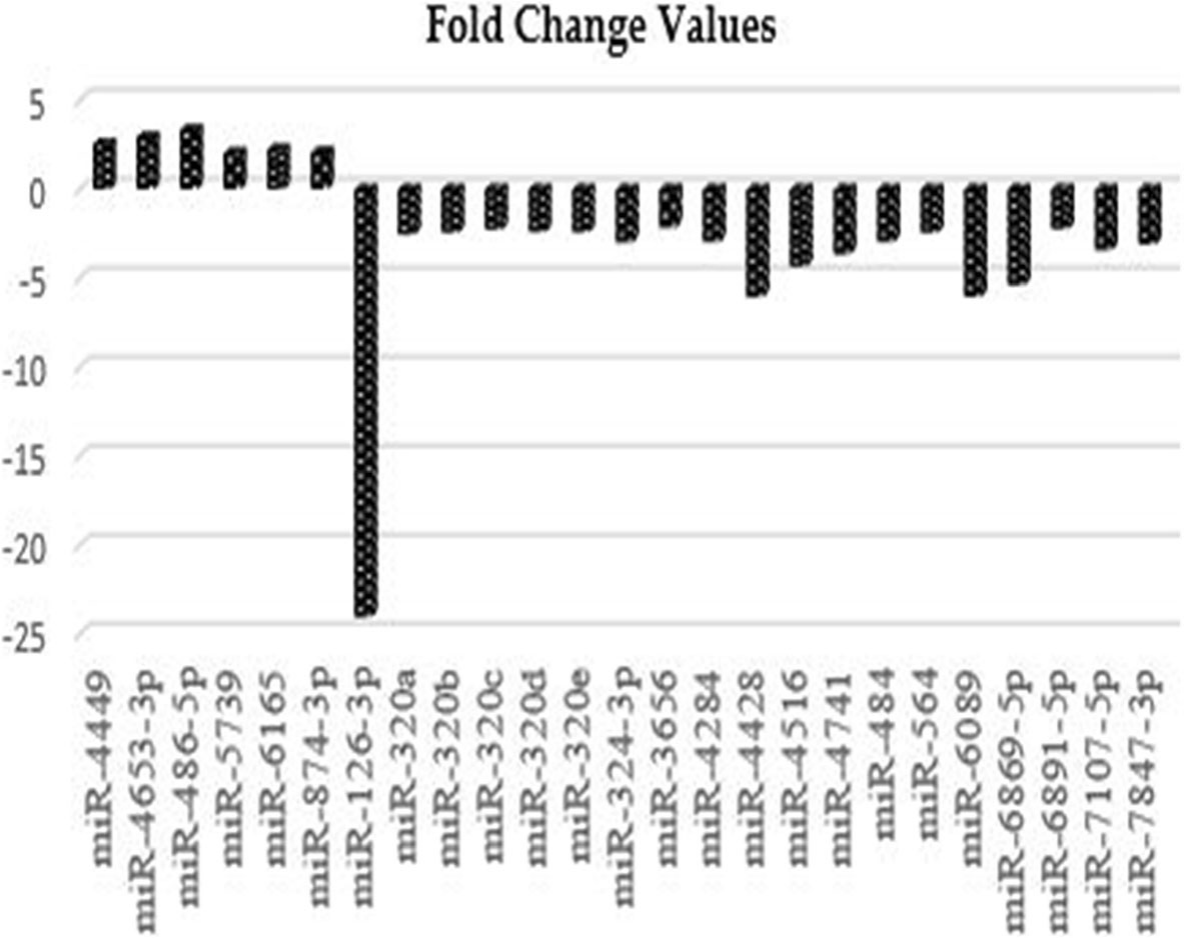
The upregulated and downregulated miRNAs and fold&changes associated with BRCA1 gene mutation positivity

Different miRNAs level were compared between group 2 in order to determine the effect of BRCA1 gene mutation. Group 2 was consisted after the comparison of the BRCA1 gene mutation carrier family members and individuals not carrying BRCA1/2 gene mutation according to miRNAs expression profiles. After the comparisons, downregulated and upregulated miRNAs related to BRCA1 gene mutation carrier were determined. The expression of a total of 6 miRNAs including miR-4449, miR-4653-3p, miR-486-5p, miR-5739, miR-6165, and miR − 874-3p associated with the BRCA1 gene mutation carrying were upregulated, and the expression of a total of 19 miRNAs including miR-126-3p, miR-320a, miR-320b, miR-320c, miR-320d, miR-320e, miR-324-3p, miR-3656, miR − 4284, miR-4428, miR-4516, miR-4741, miR-484, miR-564, miR-6089, miR-6869-5p, miR-6891-5p, miR-7107-5p and miR-7847-3p were found downregulated. After the bioinformatic analysis, a total of 25 upregulated and downregulated statistically significant miRNAs and their target molecules are shown in Table 3 and Fig. 4.

**Table 3:** BRCA1 gene mutation positivity related upregulated and downregulated miRNAs and target molecules. An additional table file shows this in more detail [see Additional file 1].

miRNAs	Fold change (FC) values	Sequence of miRNA	miRNAStatus	Target genes
miR-4449	2,56	CGUCCCGGGGCUGCGCGAGGCA	Upregulated	*ZFHX3*
miR-4653-3p	2,92	UGGAGUUAAGGGUUGCUUGGAGA	Upregulated	*ATG2A, CREBL2, MAT2A, FRS2, TMED4, UBN2*
miR-486-5p	3,34	UCCUGUACUGAGCUGCCCCGAG	Upregulated	*OLFM4,CD40,ARHGAP5, IGF1R, DOCK3, CADM1*
miR-5739	2,03	GCGGAGAGAGAAUGGGGAGC	Upregulated	*DLX6,CD207,CHIC1,PPL2A, PLXDC1*
miR-6165	2,24	CAGCAGGAGGUGAGGGGAG	Upregulated	*PER1,TFAP2A,FADS1, AMER1,LUZP1, COX6B1*
miR-874-3p	2,08	CUGCCCUGGCCCGAGGGACCGA	Upregulated	*HDAC1, AQP3, STAT3, CDK9*
miR-126-3p	−24,03	UCGUACCGUGAGUAAUAAUGCG	Downregulated	*TOM1, CRK, VEGFA, SOX2, TWF1, PITPNC1, IGFBP2, KRAS*
miR-320a	−2,55	AAAAGCUGGGUUGAGAGGGCGA	Downregulated	*MCL1, BANP, ITGB3, BMI1, NRP1, NFATC3, TRPC5*
miR-320b	−2,45	AAAAGCUGGGUUGAGAGGGCAA	Downregulated	*CDK6,DCTN5,SYNCRIP,ARF1, BCL9L, ZNF600*
miR-320c	−2,26	AAAAGCUGGGUUGAGAGGGU	Downregulated	*SYNCRIP,FBXO28,SMARCC, NPM3*
miR-320d	−2,39	AAAAGCUGGGUUGAGAGGA	Downregulated	*DCTN5, SYNCRIP, FBXO28*
miR-320e	−2,42	AAAGCUGGGUUGAGAAGG	Downregulated	*DCTN5, NPM3, ZNF275, DDX19A, NCAPD2, TXNL1*
miR-324-3p	−2,98	ACUGCCCCAGGUGCUGCUGG	Downregulated	*WNT9B, CREBBP, DVL2, WNT2B*
miR-3656	−2,16	GGCGGGUGCGGGGGUGG	Downregulated	*MRPL12, LSP1, MNT, PRDM2,* *ZNF770, CECR1*
miR-4284	−2,96	GGGCUCACAUCACCCCAU	Downregulated	*BCL2L11,RBBP5,HNRNPA1, ZNF264, TRIB3, CRTAP*
miR-4428	−6,05	CAAGGAGACGGGAACAUGGAGC	Downregulated	*MSL1,MAPRE3,MYH14,CASP2, CCND2, CDK14,TP63*
miR − 4516	-4,34	GGGAGAAGGGUCGGGGC	Downregulated	*STAT3,M6PR,GPR137C,CCND2, CCNT1, CDKN1A, SCOC, TP53*
miR-4741	−3,65	CGGGCUGUCCGGAGGGGUCGGCU	Downregulated	*DDX39B,MAPK1, ZBTB39, HMGA1,*
miR-484	−2,97	UCAGGCUCAGUCCCCUCCCGAU	Downregulated	*FIS1, PAGR1, ZEB1, SLC11A2,* *SMAD2, ANAPC7, TBRG1*
miR-564	−2,45	AGGCACGGUGUCAGCAGGC	Downregulated	*GID4, CNBP, E2F3, RCAN3, AKT2, APPL1, SLC1A2, GPR155*
miR − 6089	-6,03	GGAGGCCGGGGUGGGGCGGGGCGG	Downregulated	*NKX2, TPT1, KCTD5, BBX, SGCD, CDH7, CCNB1,*
miR-6869 − 5p	-5,38	GUGAGUAGUGGCGCGCGGCGGC	Downregulated	*TUBB2A,MAPK1,NRBF2, WEE1,HMGA2, MAPK1, STAG2*
miR-6891-5p	−2,23	UAAGGAGGGGGAUGAGGGG	Downregulated	*CHD4, CD207, DDX6, CHRDL1,* *CCND2, TP63*
miR-7107-5p	−3,43	UCGGCCUGGGGAGGAGGAAGGG	Downregulated	*VAV3, CASP16, CCND1, CASP16, MAPK14,*
miR-7847 − 3p	-3,1	CGUGGAGGACGAGGAGGAGGC	Downregulated	*HAVCR1, POTED, DNAJC10, SOD2, M6PR, CDK19*

**Fig. 5:**
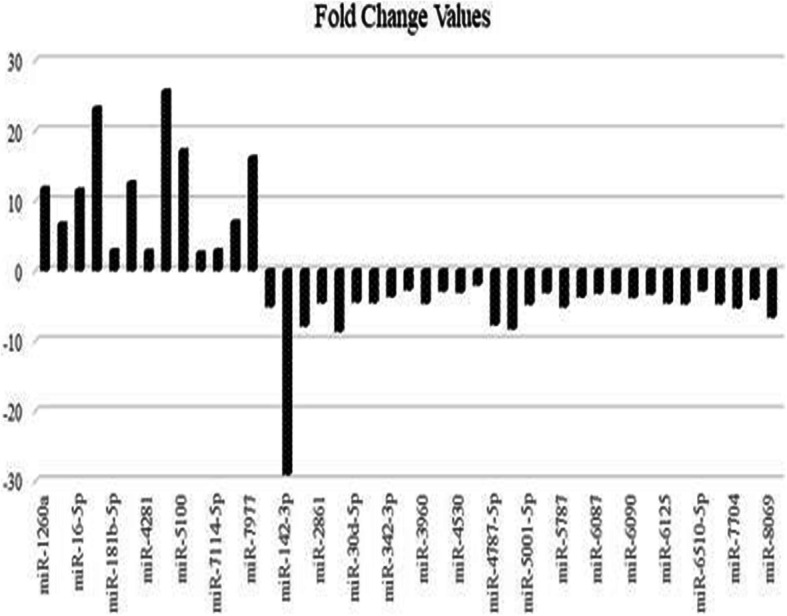
The upregulated and downregulated miRNAs and fold-changes associated with BRCA1 mutation carriage and epithelial ovarian cancer etiology

Different miRNA levels were compared between group 3 in order to determine the relation with ovarian cancer development and BRCA positivity. Group 3 consists of comparison of miRNAs of BRCA1 positive ovarian cancer patient with all other BRCA1 positive healthy individuals. After comparison upregulated and downregulated miRNAs related to mutation carriage in BRCA1 gene and epithelial ovarian cancer etiology were determined. The expression of 13 miRNAs including miR-1260a, miR-1260b, miR-16-5p, miR-17-5p, miR-181b-5p, miR − 26b-5p, miR-4281, miR-4286, miR-5100, miR-6840-3p, miR-7114-5p, miR-7975 and miR-7977 were found to have upregulated, and the expression of 30 miRNAs including miR-1225-5p, miR-142-3p, miR − 26a-5p, miR − 2861, miR-29a-3p, miR-30d-5p, miR-3196, miR-342-3p, miR-3665, miR-3960, miR-4466, miR-4530, miR-4687-3p, miR-4787-5p, miR-494-3p, miR-5001-5p, miR-5006-5p, miR-5787, miR-6068, miR-6087, miR-6088, miR-6090, miR-6124, miR-6125, miR-638, miR-6510-5p, miR-6800-5p, miR-7704, miR-8063 and miR-8069 were found to have downregulated. After bioinformatic analysis, a total of 43 upregulated and downregulated statistically significant miRNAs and the target molecules are given in Table 4 and Fig. 5.

**Table 4:** BRCA1 mutation carriage and epithelial ovarian cancer etiology related upregulated and downregulated miRNAs, target molecules. An additional table file shows this in more detail [see Additional file 2].

miRNAs	Fold change (FC) values	Sequence of miRNA	miRNAStatus	Target genes
miR-1260a	11,7	AUCCCACCUCUGCCACCA	Upregulated	*PSAT1,UNC13A, RPS27, BRD7*
miR-1260b	6,65	AUCCCACCACUGCCACCAU	Upregulated	*SFRP1, DKK2, SMAD4, PSAT1,UNC13A, RPS27*
miR-16 − 5p	11,5	UAGCAGCACGUAAAUAUUGGCG	Upregulated	*CCNE1, ARL2, BCL2, HMGA1, CDK6, CCND1, VEGFA, RECK, PRDM4*
miR-17-5p	23,2	CAAAGUGCUUACAGUGCAGGUAG	Upregulated	*TGFBR2, PTEN, CDKN1A, BCL2L11,* *E2F1,TP53,STAT3*
miR-181b-5p	2,85	AACAUUCAUUGCUGUCGGUGGGU	Upregulated	*TCL1A, TIMP3, PLAG1, BCL2,RNF2,VSNL1, ATM*
miR-26b-5p	12,5	UUCAAGUAAUUCAGGAUAGGU	Upregulated	*PTGS2, EPHA2, CHORDC1, EZH2,* *CCNE1,ABCA1, GATA4*
miR-4281	2,75	GGGUCCCGGGGAGGGGGG	Upregulated	*NCDN, CDKN1A, BCL3*
miR-4286	25,6	ACCCCACUCCUGGUACC	Upregulated	*LDLR, ZNF354B, NSD1, RABGAP1,* *TAOK1, MKNK2*
miR-5100	17,1	UUCAGAUCCCAGCGGUGCCUCU	Upregulated	*COX10, DEK, KCNN3, RAB11FIP1,* *DYNLT1, NOTCH2*
miR-6840-3p	2,51	GCCCAGGACUUUGUGCGGGGUG	Upregulated	*SLFN12L, CTC1, GXYLT2,* *GDE1,FADS1,PER1, ATG9A*
miR-7114-5p	2,89	UCUGUGGAGUGGGGUGCCUGU	Upregulated	*M6PR, HNRNPUL1, SHMT1, ZNF529,ACVR2B, PAICS, TAF8*
miR-7975	6,94	AUCCUAGUCACGGCACCA	Upregulated	*KBTBD8,GULP1, CASZ1, RAD51*
miR-7977	16,1	UUCCCAGCCAACGCACCA	Upregulated	*HSPA1B, ZNF703, TMEM185B,* *SF3B3,COX6B1, CCDC9, CDH7*
miR-1225-5p	-5,12	GUGGGUACGGCCCAGUGGGGGG	Downregulated	*ORC4, ODF2L, MTRNR2L7,* *PSMG2, MTRNR2L3*
miR-142-3p	−29,06	UGUAGUGUUUCCUACUUUAUGGA	Downregulated	*ARNTL,TGFBR1, RAC1, ROCK2,, CCNT2, TAB2, PTPN23,*
miR-26a-5p	−7,88	UUCAAGUAAUCCAGGAUAGGCU	Downregulated	*EZH2,RB1,ADAM17, HMGA2,CCND2, CPEB3, DNMT3B*
miR-2861	−4,57	GGGGCCUGGCGGUGGGCGG	Downregulated	*LY6E,CCDC64, CCND1, DARS2, APAF1*
miR-29a-3p	−8,67	UAGCACCAUCUGAAAUCGGUUA	Downregulated	*MCL1,CDK6,SPARC, DNMT3A,DNMT3B, COL4A1*
miR-30d-5p	−4,45	UGUAAACAUCCCCGACUGGAAG	Downregulated	*GNAI2, TP53, CASP3, SNAI1, EZH2, BCL9, NOTCH1, SMAD1*
miR-3196	−4,55	CGGGGCGGCAGGGGCCUC	Downregulated	*POU3F3,TULP1, H2AFX,PCGF3, CASP16,ATG2A, CCDC64*
miR − 342-3p	-3,7	UCUCACACAGAAAUCGCACCCGU	Downregulated	*GEMIN4, DNMT1, ID4, SREBF1,SREBF2, BMP7, RMND5A*
miR-3665	−2,75	AGCAGGUGCGGGGCGGCG	Downregulated	*RAB5C,DNAJC15, ZNF85, MRPL17, ELF4, ENPP6, CASP2*
miR-3960	−4,64	GGCGGCGGCGGAGGCGGGGG	Downregulated	*POU3F3,PRX,PIAS4, PEG10*
miR-4466	−2,9	GGGUGCGGGCCGGCGGGG	Downregulated	*NAGK,NFX1,F2R, DDA1, AACS*
miR-4530	−3,06	CCCAGCAGGACGGGAGCG	Downregulated	*HES4, DMPK, CALM2, GPRC5A,ATAT1, CALM2, PTCH1*
miR-4687-3p	−2,01	UGGCUGUUGGAGGGGGCAGGC	Downregulated	*ZBTB39, SLC37A4, ADAP1, FHL2,* *BARHL1, AKAP6,*
miR-4787-5p	−7,7	GCGGGGGUGGCGGCGGCAUCCC	Downregulated	*STMN3,VPS51, MYADM,CYP2B6, SNX19, PER3, FN3K*
miR-494-3p	−8,32	UGAAACAUACACGGGAAACCUC	Downregulated	*PTEN,CDK6,MYC, BCL2L11,BCL2, ATXN1, MAPK1, MDM4*
miR-5001-5p	−4,8	AGGGCUGGACUCAGCGGCGGAGCU	Downregulated	*SPTBN2,RAD54L2, NF2,CDK2, COX6B1, MAP3K9*
miR-5006-5p	−3,1	UUGCCAGGGCAGGAGGUGGAA	Downregulated	*KLHL15,ZNF354B, GIGYF1,ACTG1, RRP7A, PRPF40A*
miR − 5787	-5,16	GGGCUGGGGCGCGGGGAGGU	Downregulated	*ELF5,RAB5B,ORC4, CD4,MSH5,NF2, PARP2, PTEN*
miR-6068	−3,75	CCUGCGAGUCUCCGGCGGUGG	Downregulated	*BTG1,DUSP3, TMEM170A*
miR-6087	−3,23	UGAGGCGGGGGGGCGAGC	Downregulated	*GSG2, FADS1, CNBP, AGO3,* *CSTF2, HOXD3, BAG5*
miR-6088	−3,21	AGAGAUGAAGCGGGGGGGCG	Downregulated	*USP42,SECISBP2L, RAB22A,CASP5, MAPK1*
miR-6090	−3,83	GGGGAGCGAGGGGCGGGGC	Downregulated	*AVL9, QSOX2, GNAI2, FAM43A,E2F6, MAP3K2*
miR-6124	−3,3	GGGAAAAGGAAGGGGGAGGA	Downregulated	*DNAJB9, BCAN, E2F3, RAD21,BCL2L12, CASP3, CDKN1A*
miR-6125	−4,6	GCGGAAGGCGGAGCGGCGGA	Downregulated	*HAVCR1,AEN, ZMYM1, BICD2, BCR, CASP16,*
miR-638	−4,7	AGGGAUCGCGGGCGGGUGGCGGCCU	Downregulated	*OSCP1,SP2,SOX2, HIST2H4A, HIST2H4B, BRCA1, CD4, SOD2*
miR-6510-5p	−2,82	CAGCAGGGGAGAGAGAGGAGUC	Downregulated	*ABT1,BNC2,TPM3, COX6B1,AGO1, BCL2L13, MAP2K7*
miR-6800-5p	−4,67	GUAGGUGACAGUCAGGGGCGG	Downregulated	*SERBP1,MYCBP, CDK2, DICER1*
miR-7704	−5,28	CGGGGUCGGCGGCGACGUG	Downregulated	*IFNAR1,KCNH1, SDF4, ERBB3,*
miR-8063	−4,01	UCAAAAUCAGGAGUCGGGGCUU	Downregulated	*BCL2L11,AGO3, DNAJC21, E2F2,*
miR-8069	−6,63	GGAUGGUUGGGGGCGGUCGGCGU	Downregulated	*BTG3,UBA6,RPS27,CCND1,CDK2AP2, DICER1, HHLA1*

For identifying the ovarian cancer predisposition, all family members were compared with ovarian cancer monozygotic twins in group 4. The upregulated or downregulated miRNAs in association with the epithelial ovarian cancer risk were identified after the comparison.

**Fig. 6:**
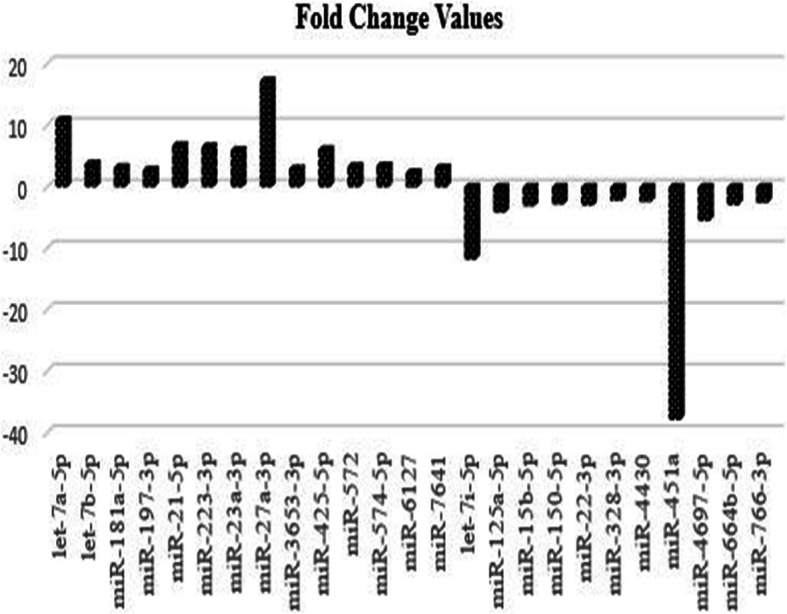
The upregulated and downregulated miRNAs and fold-changes associated with epithelial ovarian cancer predisposition

The expression of the 14 miRNAs consisting of let-7a-5p, let-7b-5p, miR-181a-5p, miR-197-3p, miR-21-5p, miR-223-3p, miR-23a-3p, miR-27a-3p, miR-3653-3p, miR-425-5p, miR-572, miR-574-5p, miR-6127, and miR-7641 were detected to be upregulated. The expression of the 11 miRNAs consisting of let-7i-5p, miR − 125a-5p, miR-15b-5p, miR-150-5p, miR-22 − 3p, miR-328-3p, miR-4430, miR-451a, miR-4697-5p, miR-664b-5p, and miR-766-3p were detected to have downregulated. A total of 25 upregulated and downregulated statistically significant miRNAs and the target molecules are given in Table 5 and Fig. 6.

**Table 5:** Epithelial ovarian cancer predisposition associated upregulated and downregulated miRNAs, target molecules. An additional table file shows this in more detail [see Additional file 3].

miRNAs	Fold change (FC) values	Sequence of miRNA	miRNAStatus	Target genes
let-7a-5p	10,8	UGAGGUAGUAGGUUGUAUAGUU	Upregulated	*NF2,KRAS,HMGA2,HMGA1,CDK6, NRAS*
let-7b-5p	3,79	UGAGGUAGUAGGUUGUGUGGUU	Upregulated	*HMGA1, IGF2BP1, CDC34, HMGA2, CDC25A, IGF2BP2*
miR-181a-5p	3,2	AACAUUCAACGCUGUCGGUGAGU	Upregulated	*CDKN1B,ATM,BCL2,KRAS, MAPK1 BRCA1,CDH13,KRAS,MMP14, NOTCH1, NOTCH2*
miR-197-3p	2,8	UUCACCACCUUCUCCACCCAGC	Upregulated	*CD82, PMAIP1, MTHFD1, RAD51,* *AGO1, CASP10, CHECK1*
miR-21-5p	6,75	UAGCUUAUCAGACUGAUGUUGA	Upregulated	*CDC25A,BCL2, PTEN, APAF1, E2F1, TP53, MSH2*
miR-223-3p	6,55	UGUCAGUUUGUCAAAUACCCCA	Upregulated	*IGF1R, PARP1 E2F1,CDK2, ATM, STAT5A, E2F1*
miR-23a-3p	5,98	AUCACAUUGCCAGGGAUUUCC	Upregulated	*MYH1,MYH2,MYH4,PTEN, APAF1, FOXO3, CDH1, MYC*
miR-27a-3p	17,2	UUCACAGUGGCUAAGUUCCGC	Upregulated	*FOXO1, SP1, APC, EGFR, CCND1,* *CDH6, NF1, NRAS, TP53, WNT9B*
miR-3653-3p	3,3	CUAAGAAGUUGACUGAAG	Upregulated	*CCND2, MYLIP, MTMR4, SMAD5, PTEN*
miR-425-5p	6,08	AAUGACACGAUCACUCCCGUUGA	Upregulated	*CCND1, PTEN, FGFR3, DICER1, E2F3, MAP2K6, NRAS*
miR-572	3,34	GUCCGCUCGGCGGUGGCCCA	Upregulated	*CDKN1A, MED29, WNT7A, ATM*
miR-574-5p	3,46	UGAGUGUGUGUGUGUGAGUGUGU	Upregulated	*FOXN3, PPP2R1B, BCL10, CCND1, CDH12, CDK15, CDKN1A, MAPK10*
miR-6127	2,32	UGAGGGAGUGGGUGGGAGG	Upregulated	*TUBB2A,MYH14, DLX6, HIP, CHRDL1*
miR-7641	3,14	UUGAUCUCGGAAGCUAAGC	Upregulated	*TRIP4,TAOK1,ARL5C,COX20, PPWD1, SLC30A4*
let-7i-5p	−11,7	UGAGGUAGUAGUUUGUGCUGUU	Downregulated	*TLR4, BMP4, EIF2C1, NEUROG1,* *SOCS1, IL13, IGF1*
miR-125a-5p	−4,11	UCCCUGAGACCCUUUAACCUGUGA	Downregulated	*ERBB3, CDKN1A, TP53, ERBB2,* *EGFR, STAT3, MYC, VEGFA*
miR-15b-5p	−3,08	UAGCAGCACAUCAUGGUUUACA	Downregulated	*BCL2,VEGFA,CCND1,CCNE1, CDK1, CDK4, CDK6, E2F3, MAPK1*
miR-150-5p	−2,77	UCUCCCAACCCUUGUACCAGUG	Downregulated	*EGR2, ZEB1, MUC4,MYB, TP53, BIRC5*
miR − 22-3p	−2,99	AAGCUGCCAGUUGAAGAACUGU	Downregulated	*CDKN1A, WNT1, ERBB3, MYCBP, HMGB1, BMP6, E2F2, PTEN*
miR-328-3p	−2,17	CUGGCCCUCUCUGCCCUUCCGU	Downregulated	*CD44, MMP16, AGO1, RAD51*
miR-4430	-2,35	AGGCUGGAGUGAGCGGAG	Downregulated	*ZNF485, ABL2, MAPK1, MSH5,PTEN*
miR-451a	−37,8	AAACCGUUACCAUUACUGAGUU	Downregulated	*CPNE3, RAB5A, IL6R, AKT1, MMP2*
miR-4697 − 5p	-5,5	AGGGGGCGCAGUCACUGACGUG	Downregulated	*SIX5, BCL7A, MEN1,VGF*
miR-664b-5p	−2,89	UGGGCUAAGGGAGAUGAUUGGGUA	Downregulated	*CD55, MSN, RHOBTB3, PLAG1*
miR-766-3p	−2,5	ACUCCAGCCCCACAGCCUCAGC	Downregulated	*COX1, MAPK1, NF2, RAD51,* *STK4, STK24, VEGFC*

## Discussion

Women are diagnosed with ovarian cancer at an advanced stage due to limited number of biological markers for ovarian cancer patients. Although existing ovarian cancer biomarkers, cancer antigen-125 and cancer antigen-15-3 (CA125, CA15–3) are sensitive in the follow-up of diagnosed gynecological cancers, they have less sensitivity in the diagnosis of early stage gynecological cancers and separation of malignant tumor formations from benign formations [19]. Therefore, to understand the underlying mechanisms of ovarian cancer and to explore targeting drugs and to improve new treatment protocols for ovarian malignancy, revealing significant genetic changes is necessary. The genetic and epidemiologic studies conducted on monozygotic twins are known to provide accurate and direct information about the gene and environment interaction with the disease occurrence mechanism [7]. The changes in genes that result in the occurrence of tumors such as miRNA expression level among the monozygotic twins provides information on the etiology of disease and may have a role as a biological indicator in identifying the early stage disease and in the follow up of the prognosis. We aimed to identify the non-invasive biological markers that may be used in the early diagnosis of ovarian cancer through investigating the miRNAs in the peripheral blood of monozygotic twin siblings discordant for ovarian cancer with the miRNA molecules of the other healthy members in the family. Thus that may cause less bias than the controls to be selected from the population. Ninety-nine different miRNA molecules presented in the study were detected after the comparison of monozygotic twin siblings who were discordant for ovarian cancer and with the other healthy individuals. Seventeen different miRNAs were found that could be used for detecting early diagnosis and prognosis of ovarian cancer between the monozygotic twin siblings who were discordant for ovarian cancer in our study. The association between 8 out of 17 miRNAs and ovarian carcinoma is being reported for the first time in this study. Due to the high number of newly detected miRNAs in our study, the discussion and comparison were only made between the candidate miRNAs. Although miR-197-3p, miR-1305, miR-6131, miR-3651, miR-3135b, miR-4430, miR-664b-5p and miR-766-3p have not been shown to be associated with ovarian cancer in literature but limited number of studies have suggested the association with other cancers.

Wang et al. found the elevated level of miR-197-3pin the same way as we do. The upregulated miR-197-3p expression level was shown to promote the cellular invasion, and metastasis in bladder cancer in that study. Researchers reported that LINC00312 gene was responsible for invasion and metastasis mechanisms and this gene inhibited the cellular migration, and invasion by suppressing the miR-197-3p expression. Similar results were detected in thyroid cancer in the study of Liu et al. [21, 22]. Jin et al. reported that increased expression level of miR-1305 caused pluripotent stem cells to accelerate the cell cycle G1/S transfer in addition to causing the cellular differentiation with the increased miR-1305 expression [23]. The expression levels of reduced miRNA-125a-5p and let-7i-5p found in the scope of our study have been shown to parallel with other studies in the literature. Langhe et al. suggested that let-7i-5p might be described as a diagnostic indicator in ovarian cancer [24]. The miRNA-125a-5p expression was upregulated to inhibit the cancer proliferation and migration in the in vitro study of Qin et al. in human cervical carcinomas [25] and miR-125a-5p upregulated expression level was demonstrated to inhibit the cervical cancer metastasis in cell lines [25]. In our study, statistically significant 82 miRNAs out of 99 miRNAs detected after the comparisons between the monozygotic twin with ovarian cancer and other healthy siblings were discussed here. We reported increased expression level of miR-4653-3p that had similar expression level in primary breast cancer tumors [26]. According to Zhong et.al, high expression level of miR-4653-3p was demonstrated to cause tamoxifen resistance by affecting the FRS2. They suggested up-regulated miR-4653-3p level would be possibly used as a therapeutic agent and will be effective in order to eliminate the tamoxifen resistance [26]. Ma et al. reported that upregulated miR-486-5p expression level was associated with estrogen receptor positive ovarian cancer occurrence and development was effective through OLFM4 expression [27]. We also found upregulated miR-486-5p expression in our study, and suggested that the occurrence of ovarian cancer was performed through the same pathway. In our study, miR-126-3 was found to be decreased in ovarian cancer. In parallel with our study, Fiala et al. reported the miR-126-3 expression level was important in angiogenesis, tumor growth, invasion and vascular inflammation and shorten the prognosis-free survival and overall survival in metastatic colorectal cancers patients [28]. The decreased miR-126-3 expression level was suggested to possibly show the same effect in ovarian cancer. Here in this study, miR-320b, miR-320c, miR-320d and miR-320e-miRNAs belonging to the miR-320 family have low expression levels in ovarian cancer were reported to found downregulated in colorectal adenomas and carcinomas by different researchers. In this study, decreased expression of miR-320 family has been shown to activate cell proliferation [29, 30].

Kuo et al. reported that increased expression of miR-324 family suppresses the growth and invasion of cells in breast cancer and suppresses cell proliferation in colorectal cancer and has been shown to have tumor suppressor effect in terms of cancer prevention [30]. We detected the decreased miR-324-3p expression level in our study. This result was in parallel with the tumor suppressor effect of this miRNA, and we suggest that might have a role in the development of ovarian cancer.

Yang et al. showed the increased expression of miR-4284 in human glioblastoma cancer stem cells reduced cell viability and induced apoptosis via the JNK / AP-1 signaling pathway [31] and in another study, it was argued that decreased expression of miR-4284 causes cervical cancer [32]. In our study, it is thought that the decreased expression level of miR-4284 can trigger the formation of epithelial ovarian cancer via the same signaling pathway shown in glioblastoma and cervical cancers.

The upregulated miR-4516 expression level caused the tumor suppression by changing the regulation of STAT3, which is the target molecule of miR-4516 and causing the decrease of vascular endothelial growth factor (VEGF), the target of STAT3 in the in vivo study of Chowdhari et al. [33]. However, we found downregulated miR-4516 expression level in our study. In contrary to the results, the down-regulated miR-4516 expression level in our study was suggested to result in VEGF increase and might be effective in the development of malignant ovarian formation.

Hu et al. in the study with cervical cancer cells suggested that miR-484 targets the ZEB1 and SMAD2 functioning as tumor suppressor, suppress the cell proliferation and epithelial mesenchymal transition and therefore might be a biologic indicator for cervix cancer [34]. In our study, it is thought that decreased miR-484 expression level detected in ovarian cancer promotes ovarian cancer development by increasing cell proliferation and it may be a novel biological marker in ovarian cancer. Mutlu et al. reported in their study on breast cancer cell lines that miR-564 directly affects the PI3K and MAPK pathways through AKT2, GNA12, GYS1 and SRF molecules and inhibit the cell cycle in G1 phase and through this pathway inhibited the proliferation and invasion of the breast cancer cells [35]. Decreased miR-564 expression was detected in ovarian cancer in our study. This result in parallel with the study results in the literature suggested that miRNA molecule increased the cell proliferation, invasion in ovarian cancer and stimulated the development of the ovarian cancer. The increased miR-1260 expression level in parallel with our study was found to increase in colorectal cancers [36], in non-small cell lung cancer [37], kidney cancers [38] and this increase was associated with the lymph node metastasis, venous invasion and was emphasized that that might be evaluated as a potential prognostic biological indicator in these cancers. The miR-5100 found upregulated in our study was reported to increase tumor expression in lung cancer by targeting Rab6 molecule [39]. The miR-5100 may have the same effect in ovarian cancer. Lower miR-1225-5p expression level detected in our study was shown to demonstrate more aggressive phenotype and strong correlation with poor prognosis in gastric cancers, and also supported the cell proliferation, colonial formation, in vitro invasion, tumor growth, and metastasis in mice [40].

Decreased miR-142-3p expression level detected in our study was reported to be significantly associated with the advanced tumor stage, lymph node metastasis and cervical invasion [41]. In addition, researchers reported that increased miR-142-3p expression level might inhibit the tumor progression, and invasion in hepatocellular carcinoma tissues [42]. We showed that miR-638 demonstrating decreased expression profile was among one of the miRNAs whose expression levels highly decreased and targeted the BRCA1.

In addition, this miRNA was found to have an important role in triple negative breast cancer progression by disrupting the BRCA1. Therefore, miR-638 was reported to be a potential prognostic biologic marker in breast cancer, and might function as a therapeutic target role in literature [43]. It is thought that this situation may also apply to ovarian cancer according to the result found in our study.

## Conclusions

miRNAs are found in all eukaryotic cells and involve in the conversion of genes into proteins and is considered as an important cause of cancer in recent years. Here in this study, after investigation of ovarian cancer monozygotic discordant twins and their healthy family members 99 miRNAs were identified for the first time. miRNAs detected in different cancer types in the literature were parallel with the miRNA expression levels in our study. There is a need for validation of 8 miRNAs, miR-197-3p, miR-1305, miR-6131, miR-3651, miR-3135b, miR-4430, miR-664b-5p and miR-766-3p, which were particularly detected between monozygotic twins and its association with ovarian cancer, were emphasized in our study in large cohorts including the ovarian cancer patients and healthy individuals. Since the study was conducted on peripheral blood miRNAs of monozygotic discordant twins and their healthy family members, it will make a contribution to the literature. We suggest 8 novel miRNAs, 197-3p, miR-1305, miR-6131, miR-3651, miR-3135b, miR-4430, miR-664b-5p and miR-766-3p and their association with ovarian cancer indicating that these molecules might be used as candidate biomarkers in the early diagnosis and the follow of treatment in ovarian cancer. In future studies, the investigation and comparison of these miRNAs with large population in the peripheral blood samples of the patients with ovarian cancer, benign ovarian disease, and healthy controls will determine the biomarker potential and potential therapeutic target. We also suggested new candidate upregulated or downregulated miRNAs, whose expression were affected by BRCA1 mutation in ovarian cancer development, might be used as a biomarker. The miRNAs have the potential non-invasive biomarker characteristics for epithelial ovarian cancer after the validations and correlations will be performed in future.

## Supplementary information

**Additional file 1.** BRCA1 gene mutation positivity related upregulated and downregulated miRNAs and target molecules.

**Additional file 2.** BRCA1 mutation carriage and epithelial ovarian cancer etiology related upregulated and downregulated miRNAs, target molecules.

**Additional file 3.** Epithelial ovarian cancer predisposition associated upregulated and downregulated miRNAs, target molecules.

## Data Availability

The datasets during and/or analysed during the current study available from the corresponding author on reasonable request.

## References

[1] Lalwani N, Prasad SR, Vikram R, Shanbhogue AK, Huettner PC, Fasih N (2011). Histologic, molecular, and cytogenetic features of ovarian cancers: implications for diagnosis and treatment. Radiographics..

[2] [Available from: http://gco.iarc.fr/today/data/factsheets/populations/792-turkey-fact-sheets.pdf.

[3] Chen VW, Ruiz B, Killeen JL, Cote TR, Wu XC, Correa CN (2003). Pathology and classification of ovarian tumors. Cancer..

[4] Gagnon A, Ye B (2008). Discovery and application of protein biomarkers for ovarian cancer. Curr Opin Obstet Gynecol.

[5] Gilks CB, Prat J (2009). Ovarian carcinoma pathology and genetics: recent advances. Hum Pathol.

[6] Kuchenbaecker KB, Hopper JL, Barnes DR, Phillips KA, Mooij TM, Roos-Blom MJ (2017). Risks of breast, ovarian, and contralateral breast cancer for BRCA1 and BRCA2 mutation carriers. JAMA..

[7] Czyz W, Morahan JM, Ebers GC, Ramagopalan SV (2012). Genetic, environmental and stochastic factors in monozygotic twin discordance with a focus on epigenetic differences. BMC Med.

[8] Zwijnenburg PJ, Meijers-Heijboer H, Boomsma DI (2010). Identical but not the same: the value of discordant monozygotic twins in genetic research. Am J Med Genet B Neuropsychiatr Genet.

[9] Martin N, Boomsma D, Machin G (1997). A twin-pronged attack on complex traits. Nat Genet.

[10] Lewis BP, Burge CB, Bartel DP (2005). Conserved seed pairing, often flanked by adenosines, indicates that thousands of human genes are microRNA targets. Cell..

[11] Bartel DP (2004). MicroRNAs: genomics, biogenesis, mechanism, and function. Cell..

[12] Liu J (2008). Control of protein synthesis and mRNA degradation by microRNAs. Curr Opin Cell Biol.

[13] Tsuchiya S, Okuno Y, Tsujimoto G (2006). MicroRNA: biogenetic and functional mechanisms and involvements in cell differentiation and cancer. J Pharmacol Sci.

[14] Scaria V, Hariharan M, Maiti S, Pillai B, Brahmachari SK (2006). Host-virus interaction: a new role for microRNAs. Retrovirology..

[15] Tzur G, Israel A, Levy A, Benjamin H, Meiri E, Shufaro Y (2009). Comprehensive gene and microRNA expression profiling reveals a role for microRNAs in human liver development. PLoS One.

[16] Calin GA, Sevignani C, Dumitru CD, Hyslop T, Noch E, Yendamuri S (2004). Human microRNA genes are frequently located at fragile sites and genomic regions involved in cancers. Proc Natl Acad Sci U S A.

[17] Sevignani C, Calin GA, Nnadi SC, Shimizu M, Davuluri RV, Hyslop T (2007). MicroRNA genes are frequently located near mouse cancer susceptibility loci. Proc Natl Acad Sci U S A.

[18] Calin GA, Croce CM (2007). Chromosomal rearrangements and microRNAs: a new cancer link with clinical implications. J Clin Invest.

[19] Jacobs IJ, Menon U (2004). Progress and challenges in screening for early detection of ovarian cancer. Mol Cell Proteomics.

[20] Penyige A, Marton E, Soltesz B, Szilagyi-Bonizs M, Poka R, Lukacs J (2019). Circulating miRNA profiling in plasma samples of ovarian cancer patients. Int J Mol Sci.

[21] Wang YY, Wu ZY, Wang GC, Liu K, Niu XB, Gu S (2016). LINC00312 inhibits the migration and invasion of bladder cancer cells by targeting miR-197-3p. Tumour Biol.

[22] Liu K, Huang W, Yan DQ, Luo Q, Min X. Overexpression of long intergenic noncoding RNA LINC00312 inhibits the invasion and migration of thyroid cancer cells by down-regulating microRNA-197-3p. Biosci Rep. 2017;37(4). 10.1042/BSR20170109 PMC5518463 28539331

[23] Jin S, Collin J, Zhu L, Montaner D, Armstrong L, Neganova I (2016). A novel role for miR-1305 in regulation of Pluripotency-differentiation balance, cell cycle, and apoptosis in human pluripotent stem cells. Stem Cells.

[24] Langhe R, Norris L, Saadeh FA, Blackshields G, Varley R, Harrison A (2015). A novel serum microRNA panel to discriminate benign from malignant ovarian disease. Cancer Lett.

[25] Qin X, Wan Y, Wang S, Xue M (2016). MicroRNA-125a-5p modulates human cervical carcinoma proliferation and migration by targeting ABL2. Drug Des Devel Ther.

[26] Zhong X, Xie G, Zhang Z, Wang Z, Wang Y, Wang Y (2016). MiR-4653-3p and its target gene FRS2 are prognostic biomarkers for hormone receptor positive breast cancer patients receiving tamoxifen as adjuvant endocrine therapy. Oncotarget..

[27] Ma H, Tian T, Liang S, Liu X, Shen H, Xia M (2016). Estrogen receptor-mediated miR-486-5p regulation of OLFM4 expression in ovarian cancer. Oncotarget..

[28] Fiala O, Pitule P, Hosek P, Liska V, Sorejs O, Bruha J (2017). The association of miR-126-3p, miR-126-5p and miR-664-3p expression profiles with outcomes of patients with metastatic colorectal cancer treated with bevacizumab. Tumour Biol.

[29] Tadano T, Kakuta Y, Hamada S, Shimodaira Y, Kuroha M, Kawakami Y (2016). MicroRNA-320 family is downregulated in colorectal adenoma and affects tumor proliferation by targeting CDK6. World J Gastrointest Oncol.

[30] Wang J, Shi C, Wang J, Cao L, Zhong L, Wang D (2017). MicroRNA-320a is downregulated in non-small cell lung cancer and suppresses tumor cell growth and invasion by directly targeting insulin-like growth factor 1 receptor. Oncol Lett.

[31] Yang F, Nam S, Brown CE, Zhao R, Starr R, Ma Y (2014). A novel berbamine derivative inhibits cell viability and induces apoptosis in cancer stem-like cells of human glioblastoma, via up-regulation of miRNA-4284 and JNK/AP-1 signaling. PLoS One.

[32] Pardini B, De Maria D, Francavilla A, Di Gaetano C, Ronco G, Naccarati A (2018). MicroRNAs as markers of progression in cervical cancer: a systematic review. BMC Cancer.

[33] Chowdhari S, Saini N (2014). hsa-miR-4516 mediated downregulation of STAT3/CDK6/UBE2N plays a role in PUVA induced apoptosis in keratinocytes. J Cell Physiol.

[34] Hu Y, Xie H, Liu Y, Liu W, Liu M, Tang H (2017). miR-484 suppresses proliferation and epithelial-mesenchymal transition by targeting ZEB1 and SMAD2 in cervical cancer cells. Cancer Cell Int.

[35] Mutlu M, Saatci O, Ansari SA, Yurdusev E, Shehwana H, Konu O (2016). miR-564 acts as a dual inhibitor of PI3K and MAPK signaling networks and inhibits proliferation and invasion in breast cancer. Sci Rep.

[36] Liu DR, Guan QL, Gao MT, Jiang L, Kang HX (2016). miR-1260b is a potential prognostic biomarker in colorectal cancer. Med Sci Monit.

[37] Xu L, Li L, Li J, Li H, Shen Q, Ping J (2015). Overexpression of miR-1260b in non-small cell lung cancer is associated with lymph node metastasis. Aging Dis.

[38] Hirata H, Ueno K, Nakajima K, Tabatabai ZL, Hinoda Y, Ishii N (2013). Genistein downregulates onco-miR-1260b and inhibits Wnt-signalling in renal cancer cells. Br J Cancer.

[39] Huang H, Jiang Y, Wang Y, Chen T, Yang L, He H (2015). miR-5100 promotes tumor growth in lung cancer by targeting Rab6. Cancer Lett.

[40] Zheng H, Zhang F, Lin X, Huang C, Zhang Y, Li Y (2016). MicroRNA-1225-5p inhibits proliferation and metastasis of gastric carcinoma through repressing insulin receptor substrate-1 and activation of beta-catenin signaling. Oncotarget..

[41] Li M, Li BY, Xia H, Jiang LL (2017). Expression of microRNA-142-3p in cervical cancer and its correlation with prognosis. Eur Rev Med Pharmacol Sci.

[42] Wu L, Cai C, Wang X, Liu M, Li X, Tang H (2011). MicroRNA-142-3p, a new regulator of RAC1, suppresses the migration and invasion of hepatocellular carcinoma cells. FEBS Lett.

[43] Tan X, Peng J, Fu Y, An S, Rezaei K, Tabbara S (2014). miR-638 mediated regulation of BRCA1 affects DNA repair and sensitivity to UV and cisplatin in triple-negative breast cancer. Breast Cancer Res.

