# Supplementary material for: miRNA expression profile changes in the peripheral blood of monozygotic discordant twins for epithelial ovarian carcinoma: potential new biomarkers for early diagnosis and prognosis of ovarian carcinoma
Source: J Ovarian Res. 2020 Aug 27;13:99. doi: 10.1186/s13048-020-00706-8 (PMC7453540; doi:10.1186/s13048-020-00706-8)
Supplement: Supplementary file 1 — Additional file 1. BRCA1 gene mutation positivity related upregulated and downregulated miRNAs and target molecules. [file 13048_2020_706_MOESM1_ESM.docx]

Table 3 BRCA1 gene mutation positivity related upregulated and downregulated miRNAs and target molecules

**miRNAs Fold Change Sequence of miRNA miRNAStatus Target Genes**

**(FC) Values**

| miR-4449 | 2,56 | CGUCCCGGGGCUGCGCGAGGCA | Upregulated | *ZFHX3* |
| --- | --- | --- | --- | --- |
| miR-4653-3p | 2,92 | UGGAGUUAAGGGUUGCUUGGAGA | Upregulated | *ATG2A, CREBL2, MAT2A, FRS2, TMED4, UBN2* |
| miR-486-5p | 3,34 | UCCUGUACUGAGCUGCCCCGAG | Upregulated | *OLFM4,CD40,ARHGAP5, IGF1R, DOCK3, CADM1* |
| miR-5739 | 2,03 | GCGGAGAGAGAAUGGGGAGC | Upregulated | *DLX6,CD207,CHIC1,PPL2A, PLXDC1* |
| miR-6165 | 2,24 | CAGCAGGAGGUGAGGGGAG | Upregulated | *PER1,TFAP2A,FADS1, AMER1,LUZP1, COX6B1* |
| miR-874-3p | 2,08 | CUGCCCUGGCCCGAGGGACCGA | Upregulated | *HDAC1, AQP3, STAT3, CDK9* |
| miR-126-3p | -24,03 | UCGUACCGUGAGUAAUAAUGCG | Downregulated | *TOM1, CRK, VEGFA, SOX2, TWF1, PITPNC1, IGFBP2, KRAS* |
| miR-320a | -2,55 | AAAAGCUGGGUUGAGAGGGCGA | Downregulated | *MCL1, BANP, ITGB3, BMI1, NRP1, NFATC3, TRPC5* |
| miR-320b | -2,45 | AAAAGCUGGGUUGAGAGGGCAA | Downregulated | *CDK6,DCTN5,SYNCRIP,ARF1, BCL9L, ZNF600* |
| miR-320c | -2,26 | AAAAGCUGGGUUGAGAGGGU | Downregulated | *SYNCRIP,FBXO28,SMARCC, NPM3* |
| miR-320d | -2,39 | AAAAGCUGGGUUGAGAGGA | Downregulated | *DCTN5, SYNCRIP, FBXO28* |
| miR-320e | -2,42 | AAAGCUGGGUUGAGAAGG | Downregulated | *DCTN5, NPM3, ZNF275, DDX19A, NCAPD2, TXNL1* |
| miR-324-3p | -2,98 | ACUGCCCCAGGUGCUGCUGG | Downregulated | *WNT9B, CREBBP, DVL2, WNT2B* |
| miR-3656 | -2,16 | GGCGGGUGCGGGGGUGG | Downregulated | *MRPL12, LSP1, MNT, PRDM2,*  *ZNF770, CECR1* |
| miR-4284 | -2,96 | GGGCUCACAUCACCCCAU | Downregulated | *BCL2L11,RBBP5,HNRNPA1, ZNF264, TRIB3, CRTAP* |
| miR-4428 | -6,05 | CAAGGAGACGGGAACAUGGAGC | Downregulated | *MSL1,MAPRE3,MYH14,CASP2, CCND2, CDK14,TP63* |
| miR-4516 | -4,34 | GGGAGAAGGGUCGGGGC | Downregulated | *STAT3,M6PR,GPR137C,CCND2, CCNT1, CDKN1A, SCOC, TP53* |
| miR-4741 | -3,65 | CGGGCUGUCCGGAGGGGUCGGCU | Downregulated | *DDX39B,MAPK1, ZBTB39, HMGA1,* |
| miR-484 | -2,97 | UCAGGCUCAGUCCCCUCCCGAU | Downregulated | *FIS1, PAGR1, ZEB1, SLC11A2,*  *SMAD2, ANAPC7, TBRG1* |
| miR-564 | -2,45 | AGGCACGGUGUCAGCAGGC | Downregulated | *GID4, CNBP, E2F3, RCAN3, AKT2, APPL1, SLC1A2, GPR155* |
| miR-6089 | -6,03 | GGAGGCCGGGGUGGGGCGGGGCGG | Downregulated | *NKX2, TPT1, KCTD5, BBX, SGCD, CDH7, CCNB1,* |
| miR-6869-5p | -5,38 | GUGAGUAGUGGCGCGCGGCGGC | Downregulated | *TUBB2A,MAPK1,NRBF2, WEE1,HMGA2, MAPK1, STAG2* |
| miR-6891-5p | -2,23 | UAAGGAGGGGGAUGAGGGG | Downregulated | *CHD4, CD207, DDX6, CHRDL1,*  *CCND2, TP63* |
| miR-7107-5p | -3,43 | UCGGCCUGGGGAGGAGGAAGGG | Downregulated | *VAV3, CASP16, CCND1, CASP16, MAPK14,* |
| miR-7847-3p | -3,1 | CGUGGAGGACGAGGAGGAGGC | Downregulated | *HAVCR1, POTED, DNAJC10, SOD2, M6PR, CDK19* |
