# Supplementary material for: miRNA expression profile changes in the peripheral blood of monozygotic discordant twins for epithelial ovarian carcinoma: potential new biomarkers for early diagnosis and prognosis of ovarian carcinoma
Source: J Ovarian Res. 2020 Aug 27;13:99. doi: 10.1186/s13048-020-00706-8 (PMC7453540; doi:10.1186/s13048-020-00706-8)
Supplement: Supplementary file 2 — Additional file 2. BRCA1 mutation carriage and epithelial ovarian cancer etiology related upregulated and downregulated miRNAs, target molecules. [file 13048_2020_706_MOESM2_ESM.docx]

Table 4 BRCA1 mutation carriage and epithelial ovarian cancer etiology related upregulated and downregulated miRNAs, target molecules

**miRNAs Fold Change Sequence of miRNA miRNAStatus Target Genes**

**(FC) Values**

| miR-1260a | 11,7 | AUCCCACCUCUGCCACCA | Upregulated | *PSAT1,UNC13A, RPS27, BRD7* |
| --- | --- | --- | --- | --- |
| miR-1260b | 6,65 | AUCCCACCACUGCCACCAU | Upregulated | *SFRP1, DKK2, SMAD4, PSAT1,UNC13A, RPS27* |
| miR-16-5p | 11,5 | UAGCAGCACGUAAAUAUUGGCG | Upregulated | *CCNE1, ARL2, BCL2, HMGA1, CDK6, CCND1, VEGFA, RECK, PRDM4* |
| miR-17-5p | 23,2 | CAAAGUGCUUACAGUGCAGGUAG | Upregulated | *TGFBR2, PTEN, CDKN1A, BCL2L11,*  *E2F1,TP53,STAT3* |
| miR-181b-5p | 2,85 | AACAUUCAUUGCUGUCGGUGGGU | Upregulated | *TCL1A, TIMP3, PLAG1, BCL2,RNF2,VSNL1, ATM* |
| miR-26b-5p | 12,5 | UUCAAGUAAUUCAGGAUAGGU | Upregulated | *PTGS2, EPHA2, CHORDC1, EZH2,*  *CCNE1,ABCA1, GATA4* |
| miR-4281 | 2,75 | GGGUCCCGGGGAGGGGGG | Upregulated | *NCDN, CDKN1A, BCL3* |
| miR-4286 | 25,6 | ACCCCACUCCUGGUACC | Upregulated | *LDLR, ZNF354B, NSD1, RABGAP1,*  *TAOK1, MKNK2* |
| miR-5100 | 17,1 | UUCAGAUCCCAGCGGUGCCUCU | Upregulated | *COX10, DEK, KCNN3, RAB11FIP1,*  *DYNLT1, NOTCH2* |
| miR-6840-3p | 2,51 | GCCCAGGACUUUGUGCGGGGUG | Upregulated | *SLFN12L, CTC1, GXYLT2,*  *GDE1,FADS1,PER1, ATG9A* |
| miR-7114-5p | 2,89 | UCUGUGGAGUGGGGUGCCUGU | Upregulated | *M6PR, HNRNPUL1, SHMT1, ZNF529,,ACVR2B, PAICS, TAF8* |
| miR-7975 | 6,94 | AUCCUAGUCACGGCACCA | Upregulated | *KBTBD8,GULP1, CASZ1, RAD51* |
| miR-7977 | 16,1 | UUCCCAGCCAACGCACCA | Upregulated | *HSPA1B, ZNF703, TMEM185B,*  *SF3B3,COX6B1, CCDC9, CDH7* |
| miR-1225-5p | -5,12 | GUGGGUACGGCCCAGUGGGGGG | Downregulated | *ORC4, ODF2L, MTRNR2L7,*  *PSMG2, MTRNR2L3* |
| miR-142-3p | -29,06 | UGUAGUGUUUCCUACUUUAUGGA | Downregulated | *ARNTL,TGFBR1, RAC1, ROCK2, , CCNT2, TAB2, PTPN23,* |
| miR-26a-5p | -7,88 | UUCAAGUAAUCCAGGAUAGGCU | Downregulated | *EZH2,RB1,ADAM17, HMGA2,CCND2, CPEB3, DNMT3B* |
| miR-2861 | -4,57 | GGGGCCUGGCGGUGGGCGG | Downregulated | *LY6E,CCDC64, CCND1, DARS2, APAF1* |
| miR-29a-3p | -8,67 | UAGCACCAUCUGAAAUCGGUUA | Downregulated | *MCL1,CDK6,SPARC, DNMT3A,DNMT3B, COL4A1* |
| miR-30d-5p | -4,45 | UGUAAACAUCCCCGACUGGAAG | Downregulated | *GNAI2, TP53, CASP3, SNAI1, EZH2, BCL9, NOTCH1, SMAD1* |
| miR-3196 | -4,55 | CGGGGCGGCAGGGGCCUC | Downregulated | *POU3F3,TULP1, H2AFX,PCGF3, CASP16,ATG2A, CCDC64* |
| miR-342-3p | -3,7 | UCUCACACAGAAAUCGCACCCGU | Downregulated | *GEMIN4, DNMT1, ID4, SREBF1,SREBF2, BMP7, RMND5A* |
| miR-3665 | -2,75 | AGCAGGUGCGGGGCGGCG | Downregulated | *RAB5C,DNAJC15, ZNF85, MRPL17, ELF4, ENPP6, CASP2* |
| miR-3960 | -4,64 | GGCGGCGGCGGAGGCGGGGG | Downregulated | *POU3F3,PRX,PIAS4, PEG10* |
| miR-4466 | -2,9 | GGGUGCGGGCCGGCGGGG | Downregulated | *NAGK,NFX1,F2R, DDA1, AACS* |
| miR-4530 | -3,06 | CCCAGCAGGACGGGAGCG | Downregulated | *HES4, DMPK, CALM2, GPRC5A,ATAT1, CALM2, PTCH1* |
| miR-4687-3p | -2,01 | UGGCUGUUGGAGGGGGCAGGC | Downregulated | *ZBTB39, SLC37A4, ADAP1, FHL2,*  *BARHL1, AKAP6,* |
| miR-4787-5p | -7,7 | GCGGGGGUGGCGGCGGCAUCCC | Downregulated | *STMN3,VPS51, MYADM,CYP2B6, SNX19, PER3, FN3K* |
| miR-494-3p | -8,32 | UGAAACAUACACGGGAAACCUC | Downregulated | *PTEN,CDK6,MYC, BCL2L11,BCL2, ATXN1, MAPK1, MDM4* |
| miR-5001-5p | -4,8 | AGGGCUGGACUCAGCGGCGGAGCU | Downregulated | *SPTBN2,RAD54L2, NF2,CDK2, COX6B1, MAP3K9* |
| miR-5006-5p | -3,1 | UUGCCAGGGCAGGAGGUGGAA | Downregulated | *KLHL15,ZNF354B, GIGYF1,ACTG1, RRP7A, PRPF40A* |
| miR-5787 | -5,16 | GGGCUGGGGCGCGGGGAGGU | Downregulated | *ELF5,RAB5B,ORC4, CD4,MSH5,NF2, PARP2, PTEN* |
| miR-6068 | -3,75 | CCUGCGAGUCUCCGGCGGUGG | Downregulated | *BTG1,DUSP3, TMEM170A* |
| miR-6087 | -3,23 | UGAGGCGGGGGGGCGAGC | Downregulated | *GSG2, FADS1, CNBP, AGO3,*  *CSTF2, HOXD3, BAG5* |
| miR-6088 | -3,21 | AGAGAUGAAGCGGGGGGGCG | Downregulated | *USP42,SECISBP2L, RAB22A,CASP5, MAPK1* |
| miR-6090 | -3,83 | GGGGAGCGAGGGGCGGGGC | Downregulated | *AVL9, QSOX2, GNAI2, FAM43A,E2F6, MAP3K2* |
| miR-6124 | -3,3 | GGGAAAAGGAAGGGGGAGGA | Downregulated | *DNAJB9, BCAN, E2F3, RAD21,BCL2L12, CASP3, CDKN1A* |
| miR-6125 | -4,6 | GCGGAAGGCGGAGCGGCGGA | Downregulated | *HAVCR1,AEN, ZMYM1, BICD2, BCR, CASP16,* |
| miR-638 | -4,7 | AGGGAUCGCGGGCGGGUGGCGGCCU | Downregulated | *OSCP1,SP2,SOX2, HIST2H4A, HIST2H4B, BRCA1, CD4, SOD2* |
| miR-6510-5p | -2,82 | CAGCAGGGGAGAGAGAGGAGUC | Downregulated | *ABT1,BNC2,TPM3, COX6B1,AGO1, BCL2L13, MAP2K7* |
| miR-6800-5p | -4,67 | GUAGGUGACAGUCAGGGGCGG | Downregulated | *SERBP1,MYCBP, CDK2, DICER1* |
| miR-7704 | -5,28 | CGGGGUCGGCGGCGACGUG | Downregulated | *IFNAR1,KCNH1, SDF4, ERBB3,* |
| miR-8063 | -4,01 | UCAAAAUCAGGAGUCGGGGCUU | Downregulated | *BCL2L11,AGO3, DNAJC21, E2F2,* |
| miR-8069 | -6,63 | GGAUGGUUGGGGGCGGUCGGCGU | Downregulated | *BTG3,UBA6,RPS27,CCND1,CDK2AP2, DICER1, HHLA1* |
