# Supplementary material for: miRNA expression profile changes in the peripheral blood of monozygotic discordant twins for epithelial ovarian carcinoma: potential new biomarkers for early diagnosis and prognosis of ovarian carcinoma
Source: J Ovarian Res. 2020 Aug 27;13:99. doi: 10.1186/s13048-020-00706-8 (PMC7453540; doi:10.1186/s13048-020-00706-8)
Supplement: Supplementary file 3 — Additional file 3. Epithelial ovarian cancer predisposition associated upregulated and downregulated miRNAs, target molecules. [file 13048_2020_706_MOESM3_ESM.docx]

Table 5 Epithelial ovarian cancer predisposition associated upregulated and downregulated miRNAs, target molecules.

**miRNAs Fold Change Sequence of miRNA miRNAStatus Target Genes**

**(FC) Values**

| let-7a-5p | 10,8 | UGAGGUAGUAGGUUGUAUAGUU | Upregulated | *NF2,KRAS,HMGA2,HMGA1,CDK6, NRAS* |
| --- | --- | --- | --- | --- |
| let-7b-5p | 3,79 | UGAGGUAGUAGGUUGUGUGGUU | Upregulated | *HMGA1, IGF2BP1, CDC34, HMGA2, CDC25A, IGF2BP2* |
| miR-181a-5p | 3,2 | AACAUUCAACGCUGUCGGUGAGU | Upregulated | *CDKN1B,ATM,BCL2,KRAS, MAPK1 BRCA1,CDH13,KRAS,MMP14, NOTCH1, NOTCH2* |
| miR-197-3p | 2,8 | UUCACCACCUUCUCCACCCAGC | Upregulated | *CD82, PMAIP1, MTHFD1, RAD51,*  *AGO1, CASP10, CHECK1* |
| miR-21-5p | 6,75 | UAGCUUAUCAGACUGAUGUUGA | Upregulated | *CDC25A,BCL2, PTEN, APAF1, E2F1, TP53, MSH2* |
| miR-223-3p | 6,55 | UGUCAGUUUGUCAAAUACCCCA | Upregulated | *IGF1R, PARP1 E2F1,CDK2, ATM, STAT5A, E2F1* |
| miR-23a-3p | 5,98 | AUCACAUUGCCAGGGAUUUCC | Upregulated | *MYH1,MYH2,MYH4,PTEN, APAF1, FOXO3, CDH1, MYC* |
| miR-27a-3p | 17,2 | UUCACAGUGGCUAAGUUCCGC | Upregulated | *FOXO1, SP1, APC, EGFR, CCND1,*  *CDH6, NF1, NRAS, TP53, WNT9B* |
| miR-3653-3p | 3,3 | CUAAGAAGUUGACUGAAG | Upregulated | *CCND2, MYLIP, MTMR4, SMAD5, PTEN* |
| miR-425-5p | 6,08 | AAUGACACGAUCACUCCCGUUGA | Upregulated | *CCND1, PTEN, FGFR3, DICER1, E2F3, MAP2K6, NRAS* |
| miR-572 | 3,34 | GUCCGCUCGGCGGUGGCCCA | Upregulated | *CDKN1A, MED29, WNT7A, ATM* |
| miR-574-5p | 3,46 | UGAGUGUGUGUGUGUGAGUGUGU | Upregulated | *FOXN3, PPP2R1B, BCL10, CCND1, CDH12, CDK15, CDKN1A, MAPK10* |
| miR-6127 | 2,32 | UGAGGGAGUGGGUGGGAGG | Upregulated | *TUBB2A,MYH14, DLX6, HIP, CHRDL1* |
| miR-7641 | 3,14 | UUGAUCUCGGAAGCUAAGC | Upregulated | *TRIP4,TAOK1,ARL5C,COX20, PPWD1, SLC30A4* |
| let-7i-5p | -11,7 | UGAGGUAGUAGUUUGUGCUGUU | Downregulated | *TLR4, BMP4, EIF2C1, NEUROG1,*  *SOCS1, IL13, IGF1* |
| miR-125a-5p | -4,11 | UCCCUGAGACCCUUUAACCUGUGA | Downregulated | *ERBB3, CDKN1A, TP53, ERBB2,*  *EGFR, STAT3, MYC, VEGFA* |
| miR-15b-5p | -3,08 | UAGCAGCACAUCAUGGUUUACA | Downregulated | *BCL2,VEGFA,CCND1,CCNE1, CDK1, CDK4, CDK6, E2F3, MAPK1* |
| miR-150-5p | -2,77 | UCUCCCAACCCUUGUACCAGUG | Downregulated | *EGR2, ZEB1, MUC4,MYB, TP53, BIRC5* |
| miR-22-3p | -2,99 | AAGCUGCCAGUUGAAGAACUGU | Downregulated | *CDKN1A, WNT1, ERBB3, MYCBP, HMGB1, BMP6, E2F2, PTEN* |
| miR-328-3p | -2,17 | CUGGCCCUCUCUGCCCUUCCGU | Downregulated | *CD44, MMP16, AGO1, RAD51* |
| miR-4430 | -2,35 | AGGCUGGAGUGAGCGGAG | Downregulated | *ZNF485, ABL2, MAPK1, MSH5,PTEN* |
| miR-451a | -37,8 | AAACCGUUACCAUUACUGAGUU | Downregulated | *CPNE3, RAB5A, IL6R, AKT1, MMP2* |
| miR-4697-5p | -5,5 | AGGGGGCGCAGUCACUGACGUG | Downregulated | *SIX5, BCL7A, MEN1,VGF* |
| miR-664b-5p | -2,89 | UGGGCUAAGGGAGAUGAUUGGGUA | Downregulated | *CD55, MSN, RHOBTB3, PLAG1* |
| miR-766-3p | -2,5 | ACUCCAGCCCCACAGCCUCAGC | Downregulated | *COX1, MAPK1, NF2, RAD51,*  *STK4, STK24, VEGFC* |
